# Shaping Orthodontics of the Future: Concepts and Implications from a Cellular and Molecular Perspective

**DOI:** 10.3390/ijms26178203

**Published:** 2025-08-23

**Authors:** Thorsten Steinberg, Britta Jung, Ayman Husari, Shuoqiu Bai, Pascal Tomakidi

**Affiliations:** 1 Center for Dental Medicine, Division of Oral Biotechnology, Medical Center-University of Freiburg, Faculty of Medicine, University of Freiburg, Hugstetterstr. 55, 79106 Freiburg, Germany; thorsten.steinberg@uniklinik-freiburg.de (T.S.); shuoqiu.bai@uniklinik-freiburg.de (S.B.); 2 Center for Dental Medicine, Department of Orthodontics, Medical Center-University of Freiburg, Faculty of Medicine, University of Freiburg, Hugstetterstr. 55, 79106 Freiburg, Germany; britta.jung@uniklinik-freiburg.de (B.J.); ayman.husari@uniklinik-freiburg.de (A.H.)

**Keywords:** mechanotransduction, mechanobiology, mechanosignaling, focal adhesion kinase, yes-associated protein, tissue homeostasis, in vitro cell system(s), aging, senescence, cancer, wound healing

## Abstract

Orthodontic tooth movement (OTM) is accompanied by sterile inflammation, a necessary biological process that facilitates tooth displacement but also contributes to adverse effects, including hyalinization and orthodontically induced external apical root resorption (OEARR). Despite advancements in orthodontic therapies, the inflammatory response—regulated by dynamic interactions between tissue-specific cells and their molecular mediators—remains a critical factor influencing treatment outcomes. This review summarizes the current understanding of the cellular and molecular mechanisms underlying OTM, with a focus on how these insights can support the development of targeted therapeutic strategies. These include cell- and molecule-based therapies, biomaterial-mediated delivery systems, and applications of artificial intelligence (AI). Notably, AI offers promising opportunities for modeling and simulating biological responses, enabling the optimization of individualized treatment planning. We further discuss current clinical practices and highlight emerging experimental findings, with an emphasis on unresolved research questions pivotal to improving therapeutic efficacy and reducing complications such as OEARR. This comprehensive overview aims to inform future directions in orthodontics by integrating mechanistic knowledge with technological innovation.

## 1. Introduction

The oral cavity harbors the periodontium, i.e., the tooth holding apparatus, which comprises the gingival epithelium and its related connective tissue, the periodontal ligament (PDL), the alveolar bone and the root cementum [1]. The teeth that are anchored within the periodontium must be positioned in such a way that they ensure correct occlusion, as malocclusion can cause problems with chewing food but can also negatively affect speech formation. Studies carried out in this regard have shown that appropriate orthodontic treatment has a positive influence on masticatory function and speech development. These issues are extensively reviewed in two recently published articles, which is why they will not be discussed in more detail here [2,3].

Regardless of whether people decide to have misaligned teeth corrected as part of orthodontic treatment for functional or aesthetic reasons, the correction of such misalignments always involves remodeling processes within the periodontal tissues, especially the PDL and the alveolar bone. Since externally applied targeted mechanical forces act on the tooth during orthodontic treatment in order to eliminate its misalignment and the correction of the misalignment is based on tissue remodeling processes, the complexity of these two aspects can actually be reduced to the minimalist approach of “mechanics meets biology”. This slogan published by Krishnan and colleagues in 2021 also shows that if one wants to understand orthodontic tooth movement (OTM), one cannot avoid dealing intensively with the cells of the periodontal tissues and their molecules, since these are the actual OTM-related control elements [4,5].

When looking at this cellular-molecular level, it becomes clear that the orthodontically applied mechanical force first addresses the PDL in addition to the alveolar bone and induces fluid shifts in these two tissues. In this context, respective gene analysis studies among others revealed the involvement of genes assigned to extracellular matrix (ECM) reorganization and inflammation [6]. Since the PDL is a very matrix-rich connective tissue, the force exposure here primarily leads to a deformation of the ECM, which, depending on the magnitude of the force, also includes matrix perturbation. In this context, collagen rupture at certain chemical bonds has selectively been described for tensile stress [7]. Matrix deformation is designed in opposite ways, since due to the direction of the mechanical forces and the resulting directionality of the tooth movement, a trailing side (tension) and a leading side (compression) arise, i.e., positive tensile forces act on one side of the periodontium, while negative strain forces act on the other side. The negative strain forces acting on the leading side of the PDL press on the PDL-innate blood vessels and hinder the perfusion of the tissue with blood. This hindrance leads to an undersupply of oxygen to the tissue and induction of autophagy as well as apoptotic cell death caused by this hypoxia [8]. That cell death is probably preceded by autophagy in affected PDL cells, with the cell death itself apparently occurs through B-cell lymphoma protein 2 (Bcl-2)-associated X (Bax) protein-mediated mitochondrial apoptosis [9]. Studies on cementoblasts have also shown that, in addition to apoptosis, cell death through necrosis also plays a role. At the molecular level, hypoxia-inducible factor 1-alpha (HIF-1α) appears to be involved in both types of cell death [10]. This aspect is discussed in more detail in the context of orthodontically induced external apical root resorption (**OEARR**) in Section 3.3.2. Due to the previously described cell death, cell-free zones arise in the PDL and the necrosis induces a bacteria-free, i.e., aseptic, inflammasome-mediated and interleukin (IL)-1β- based inflammatory reaction [11], which as a consequence involves the destruction of the ECM. OTM-related matrix degradation is carried out by special enzymes called matrix metalloproteinases (MMPs) [12], which, among other things, destroy the collagen molecules within the PDL [13]. As an example, MMP-9 exerts collagen triple helix denaturation by intramolecular conformational changes [14], thereby inactivating collagens’ biological function. This biologically inactive but still present form of collagen in combination with the cell death described above is called hyalinization. Another consequence of the negative strain force-induced closure of nerve endings and blood vessels is vasodilation in the PDL, which induces the extravasation of leukocytes [15]. These cells, which include B and T lymphocytes, monocytes, macrophages and granulocytes (neutrophils), release numerous inflammatory mediators and chemokines including interleukin-1β (IL-1β), interleukin-6 (IL-6), interleukin-8 (IL-8), tumor necrosis factor (TNF)-α, interferon (IFN)-γ, and prostaglandin E2 (PGE2) that ultimately orchestrate the bone remodeling processes that are essential for tooth movement [15,16].

Recent advances in orthodontic biology further underscore the importance of the cellular and molecular framework of OTM. One key development has been the discovery that orthodontic forces can elicit systemic immune responses mirroring the local remodeling process, suggesting a more expansive scope of sterile inflammation than previously appreciated [17]. Cross-disciplinary progress is also evident in biomaterials: for example, a novel Ni-free β-titanium alloy nanocomposite (Nb–TiNb) with a single-phase BCC nanostructure has been engineered with a yield strength (~640 MPa) far surpassing that of conventional β-Ti alloys [18]. Such high-strength, biocompatible materials hold promises for future orthodontic appliances and implants that effectively integrate mechanical performance with biological compatibility. Meanwhile, emerging therapeutic strategies aim to harness molecular biology for clinical benefit. The use of mesenchymal cell-derived exosomes as an adjunct to OTM has been shown to significantly accelerate tooth movement while attenuating local inflammation and root resorption in animal models [19]. Similarly, pharmacological enhancement of autophagy (e.g., via rapamycin) can rejuvenate the bone remodeling response in older individuals, mitigating age-related slowdowns in tooth movement [20].

Collectively, these findings reinforce that the cellular and molecular basis of OTM is not only a fundamental scientific concept but also a practical foundation for innovations that could make orthodontic treatments faster, safer, and more personalized.

Based on the cellular and molecular events just described, which are directly related to OTM, this review article aims to highlight cellular and molecular-based strategies that can enrich the existing classical and modern concepts of orthodontic treatment design in order to provide the practitioner of the future with the possibility of achieving treatment outcomes more efficiently and with as few side effects as possible. 

## 2. Methods

This review aims to explore the cellular and molecular foundations underlying OTM and to highlight current and future cellular, molecular, biomaterial, and AI-driven approaches for enhancing orthodontic therapy. To provide a comprehensive analysis, a systematic literature review was conducted, focusing on the most relevant studies from biomedicine and biomaterials, including experimental and clinically oriented molecular concepts.

The literature search primarily utilized text-based meta-databases such as the National Library of Medicine (NLM), PubMed, and other biomedical databases. Key search terms related to the cellular and molecular aspects of OTM, including the RANK-RANKL-OPG signaling system, inflammatory responses, PDL regeneration, and orthodontically induced OEARR, were used to identify relevant studies. In addition, specific search terms were applied to explore biomaterial strategies for controlled drug delivery and tissue regeneration, as well as the role of artificial intelligence in orthodontic treatment planning.

For each topic, both primary research articles and review papers were included. The inclusion of reviews was necessary to provide broader insights into complex processes, while primary research was prioritized to ensure the most up-to-date and experimentally validated findings. The search strategy encompassed the following major themes:

Cellular and Molecular Mechanisms of OTM: Focusing on the RANK-RANKL-OPG system, osteoclastogenesis, osteogenesis, and the role of cytokines and growth factors.

Inflammatory Processes During OTM: Examining the underlying mechanisms of inflammation and its contribution to tissue remodeling and side effects such as OEARR.

Regeneration of the PDL and ECM: Assessing current and prospective biomaterials and regenerative strategies, including stem cells and recombinant collagen-based therapies.

Control of Inflammation and Bone Remodeling: Investigating molecular targets, such as MMP inhibitors and small molecules like PROTACs, that can modulate inflammatory responses and promote controlled bone resorption.

Orthodontically Induced External Apical Root Resorption (OEARR): Focusing on genetic predispositions, inflammatory mediators, and therapeutic interventions like for instance RGD mimetics.

Application of AI: Exploring AI-driven systems for treatment prediction, patient-specific planning, and real-time optimization of orthodontic care.

To ensure a comprehensive understanding, the review also integrated studies on biologics, particularly monoclonal antibodies, and their potential to modulate key molecular pathways in orthodontic care. This systematic approach allowed for a critical evaluation of both experimental and clinical perspectives, ensuring that the review provides a balanced and forward-thinking analysis of the molecular-based strategies shaping the future of orthodontics.

## 3. Orthodontic Tooth Movement: Cellular and Molecular Mechanisms, Clinical Implications, and Future Perspectives for Precision Therapy and Innovation

### 3.1. Clinical and Biological Framing of Orthodontic Tooth Movement (OTM)

Orthodontic tooth movement (OTM) relies on the application of controlled mechanical forces to achieve therapeutic displacement of teeth. This biomechanical process is mediated by an adaptive response within the periodontal ligament (PDL), alveolar bone, and surrounding tissues. From a biological standpoint, OTM constitutes a form of aseptic inflammation, triggering cellular remodeling cascades that involve fibroblasts, osteoblasts, osteoclasts, endothelial cells, macrophages, and associated signaling networks [21,22,23].

Understanding the clinical implications of these responses is central to modern orthodontics. For example, differences in individual inflammatory responses contribute to variability in treatment duration and risk of adverse outcomes such as orthodontically induced external apical root resorption (OEARR). Current biological research aims to identify molecular regulators—such as cytokines, matrix metalloproteinases (MMPs), and gene polymorphisms—that influence this balance between tissue remodeling and damage.

This review focuses on elucidating these regulatory mechanisms and highlighting translational approaches that aim to optimize OTM outcomes through biologically informed interventions.

### 3.2. The Models of OTM and Pilon’s Model of the 4 Phases

Taking into account the history of orthodontics and the existing level of knowledge of OTM at the relevant times, Burstone in 1962 and Pilon in 1996 developed models that divide OTM into different phases based on their measurable processes.

While Burstone’s *OTM* model only distinguished three phases from each other in the context of the tooth movements measurable over the respective observation time, namely the (i) initial phase, (ii) lag phase and (iii) post-lag phase [24,25], Pilon et al. discriminated against four phases, the (i) initial phase (phase I), (ii) the arrest phase (phase II), (iii) the acceleration phase (phase III), and (iv) the linear phase (phase IV). Based on the time–displacement curves determined in adult male beagle dogs, phase 1 in Pilon’s model was characterized by the reduction in the width of the PDL in response to the mechanical force. This reaction is due to the viscoelastic properties of the PDL. The subsequent phase, phase 2, was characterized by an arrest of tooth movement, which, according to the authors, was an obvious consequence of the hyalinization already mentioned in the introduction. The authors emphasize that this phase can vary greatly in time, which is most likely based on the individuality of each patient and the magnitude of the force applied. The next phase, phase 3, owes its name as the acceleration phase to the very strong remodeling processes in the PDL and alveolar bone. The following phase, phase 4, is characterized by more or less constant tooth movement [26]. Moreover, in this phase bone remodeling occurs, since alveolar bone is resorbed at the leading side of the root, while formation of new bone occurs at the trailing side. For the interested reader, these aspects in connection with OTM can be found in more detail in the following review articles [4,25,27].

### 3.3. The Cellular and Molecular Processes Which Form the Basis of OTM

#### 3.3.1. OTM-Related Periodontal Stress, Cell Death, Inflammation and PDL Hyalinization

**Figure 1:**
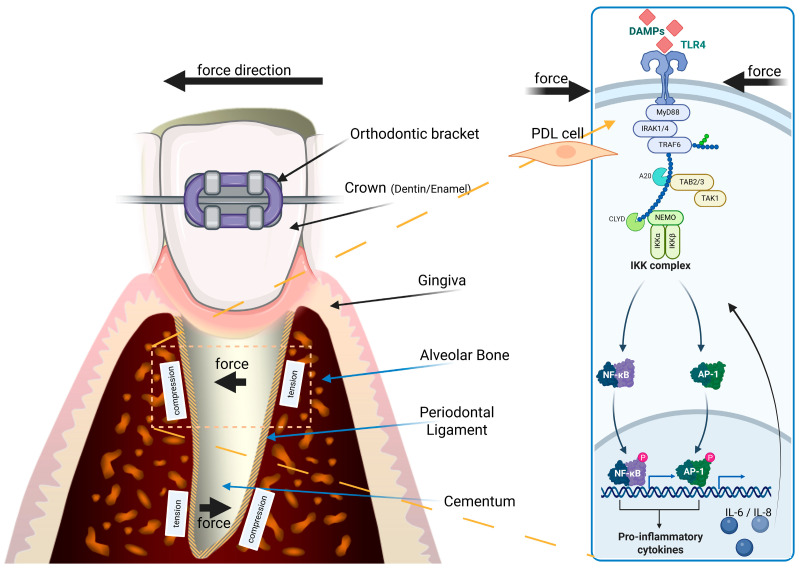
Force-induced mechanotransduction and inflammatory signaling pathways during orthodontic tooth movement. Schematic illustration of the biological and molecular mechanisms underlying orthodontic tooth movement (OTM). (**Left**) A tooth subjected to orthodontic force via an orthodontic bracket is displaced within its alveolar socket. The applied force generates distinct mechanical environments: compression on the side toward the force vector and tension on the opposite side. These forces are transmitted through the periodontal ligament (PDL), leading first to cellular responses in the respective PDL cells. (**Right**) At the molecular level, mechanical stress activates mechanoreceptors such as Toll-like receptor 4 (TLR4) in PDL cells, initiating a downstream signaling cascade involving adaptor molecules including MyD88, IRAK1/4, and TRAF6. The signal transduction is modulated by regulatory proteins such as A20 and CYLD and leads to the activation of the IKK complex (including NEMO, IKKα, and IKKβ). This activation promotes nuclear translocation of transcription factors nuclear factor kappa-B (NF-κB) and activator protein 1 (AP-1), which in turn upregulate the expression of pro-inflammatory cytokines such as IL-6 and IL-8. These cytokines contribute to tissue remodeling processes essential for tooth movement. Created in BioRender. Steinberg, T. (2025) https://BioRender.com/61so9lp.

**Figure 2:**
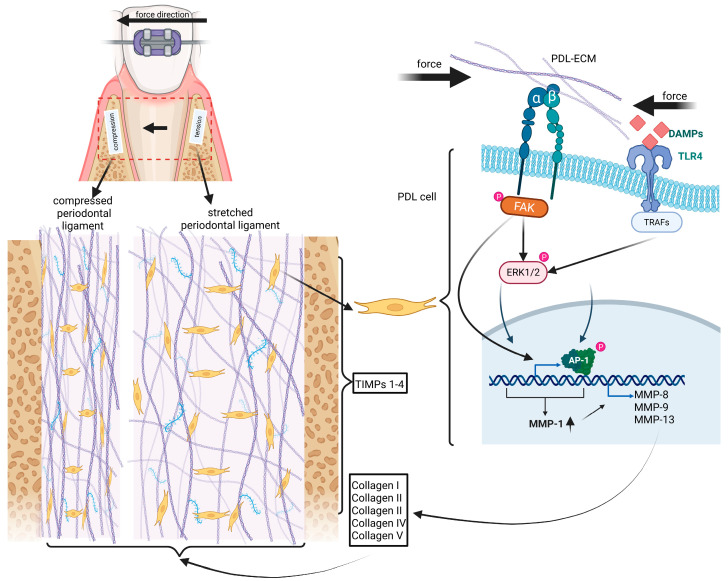
Integrin- and TLR4-dependent mechanotransduction pathways regulating matrix remodeling in the periodontal ligament during orthodontic tooth movement. Application of orthodontic force induces mechanical deformation of the periodontal ligament (PDL), resulting in areas of compression and tension. These mechanical stimuli are transduced by PDL cells via integrins (α/β subunits) and Toll-like receptor 4 (TLR4). Engagement of integrins with extracellular matrix (ECM) components initiates intracellular signaling through focal adhesion kinase (FAK) and ERK1/2 phosphorylation. Concurrently, damage-associated molecular patterns (DAMPs) activate TLR4, triggering TRAF-mediated signaling, which converges on transcriptional activation pathways. These signals lead to nuclear translocation of phosphorylated AP-1 and upregulation of matrix metalloproteinases (MMPs), including MMP-1, MMP-8, MMP-9, and MMP-13. MMPs degrade ECM components such as collagen types I, II, III, IV, and V, facilitating matrix remodeling. The process is tightly regulated by tissue inhibitors of metalloproteinases (TIMPs 1–4) to maintain tissue integrity during orthodontic tooth movement. Created in BioRender. Steinberg, T. (2025) https://BioRender.com/bse1run.

The application of mechanical forces during orthodontic treatment induces a stressful situation, especially in the PDL and the cells located therein. This stress is of a dual nature, as on the one hand it is recruited from the hypoxia on the OTM leading side and on the other hand, it is a consequence of the mechanical stress on the ECM and the associated PDL cells (Figure 1). Regarding this mechanical stress, it is worth mentioning that mechanistic studies on in vitro cultured PDL cells have shown that the mechanical stress signals are recognized and processed by TOLL-like receptors (TLRs). Particularly involved is TLR-4, activated via DAMPs (Damage-Associated Molecular Patterns) and the downstream activation of the MyD88; IRAK1/4; TRAF6-complex, resulting in NF-kB translocation into the nucleus and thereby inducing the expression of pro-inflammatory cytokines, such as IL-6 and IL-8 [28] (Figure 1 and Figure 2), demonstrating causal linkage to the mechanoperception by TLR-4 (TLR-4 belongs to the family of pattern recognition receptors [PRR]) [29,30].

Studies on patients and animal models suggest that in the context of the stressful situation, cell death occurs, in which both possibilities of cell death come into consideration, i.e., necrosis [31] as well as apoptosis [9]. In patients undergoing OTM, Kumar et al. detected significant increases in the enzymes alkaline phosphatase (ALP) and lactate dehydrogenase (LDH) within the gingival crevicular fluid (GCF). From this result, the authors concluded that under stress, the periodontium exhibits bone remodeling, as indicated by ALP increase, and cell necrosis, as indicated by LDH elevation. In addition to the significant increase in the aforementioned enzymes, the authors detected high amounts of pro-inflammatory cytokines including TNF-α, IL-1β, and IL-6 in the GCF [31]. The biological importance of these biomarkers in the context of OTM per se and with respect to GCF is reviewed in detail in d’Apuzzo et al. [32]. Wang et al. were able to show that autophagy-associated genes were initially upregulated during OTM, but that in later phases, there was a significantly increased expression of apoptosis-relevant markers such as Bax and caspase 3 [9]. In addition, studies on PDL-resident stem cells have shown that cyclic forces can induce pyroptosis, a lytic form of programmed cell death, controlled by inflammatory mediators, i.e., inflammasome-inherent caspases such as caspase-1 [33]. Caspase-1 not only regulates this process but also inflammation itself, since this protease converts the pro form of the inflammatory mediator IL-1β into the biologically active form by cleaving it [34]. The concerted action between physical stress, hypoxia, vasodilation and leukocyte extravasation as well as OTM-triggered induction of sterile (without bacterial involvement) inflammation within the periodontium has already been described in the introduction.

One consequence of the stress situation created in the PDL as a result of force application is the inflammation-related activation of MMPs, which are responsible for the already mentioned MMP-driven hyalinization, including the biofunctional inactivation of ECM molecules, especially collagen. MMPs that are repeatedly mentioned in the course of OTM are MMP-8, MMP-9 and MMP-13, which, among other things, are associated with increased expression on the leading side in animal experiments [13] (Figure 2). The substrate specificity of the aforementioned MMPs includes the fibrillar collagens of types I, II, III and V (COL1-5), each with a different preference. MMP-9 also cleaves network-forming collagens, such as collagen type IV, which is mainly involved in the formation of epithelial basement membranes [35,36,37]. In this context, studies on patients who underwent periodontal therapy in combination with OTM to eliminate malocclusion have shown that during combinatorial treatment, the detectable levels of MMP-9 in the GCF decreased significantly. Furthermore, the authors were able to show in their study that the levels of MMP-9 measurable in GCF correlated with the degree of malocclusion during orthodontic therapy [38].

The natural antagonists of MMPs are the so-called tissue inhibitors of MMPs (TIMPs), which are grouped into a family from TIMP1 to TIMP4 of which TIMPs 2 and 4 can inhibit almost all MMPs. TIMPs inhibit MMPs by binding to their catalytic domain, which determines the substrate specificity of an MMP. For example, TIMP1 binds to the catalytic domain of MMP-9 [39], which is also relevant in the OTM context. One role of TIMPs that is directly related to the control of MMP activity and that should not go unmentioned in the context of OTM as an inflammatory process is the regulation of inflammatory mediators, such as IL-1α/β or TNF-α. This is because these mediators, as well as numerous chemokines that serve as attractants of leukocytes, are converted from the pro form into the biologically active form by the proteolytic activity of MMPs [39,40].

With regard to the expression of TIMP1, in vitro studies on human periodontal ligament-derived mesenchymal stromal cells (hPDL-MSCs) have shown that the increased expression of TIMP1 induced by the inflammatory mediator IL-1β was significantly downregulated by the application of static stretching forces. From their findings, the authors concluded that low tensile forces, such as those encountered in orthodontic treatment, trigger a cell response more associated with ECM degradation [41] (Figure 2). This is because the balance or imbalance between TIMP and MMP abundance per se probably determines whether the balance settles in favor of matrix maintenance or its degradation. In a study on patients, Grant et al. were able to show that, in addition to other pro-inflammatory mediators and MMP-9, TIMPs 1 and 2 were also detectable in the GCF on the OTM trailing side. Interestingly, the inflammatory mediators and MMP-9 remained detectable on the leading side, but not for TIMP1 nor TIMP2 [42]. From the previously described in vitro experiments and patient observations, it can be assumed that orthodontic forces in the PDL can negatively influence the expression of TIMPs and that this negative influence depends on whether one focuses on the trailing or the leading side of the OTM. This assumption would also offer a possible explanation for the fact that the MMP activity on the trailing side is lower than on the leading side. This assumption together with the multitude of parameters that influence MMP and corresponding TIMP expression during orthodontic treatment are very clearly discussed in a related review article. In this review, Behm et al. come to the conclusion that there are no specific combinations of MMPs and TIMPs in treatment-related OTM, but that the expression of proteases and inhibitors is situation-dependent, that is, it depends on multiple parameters, for example, which tooth is addressed or how high the force level is or which appliance is used [43]. For a summary, see Table 1.

**Executive Summary**

Orthodontic force induces hypoxia, mechanical strain, and aseptic inflammation in the PDL, leading to various forms of cell death (apoptosis, necrosis, pyroptosis). These events trigger the release of inflammatory cytokines (e.g., IL-1β, TNF-α), MMP activation, and ECM degradation. Hyalinized zones form, delaying OTM until cleared by immune cells.

#### 3.3.2. The Phenomenon of Macrophage Polarization (MP) and Its Role in OTM-Induced OEARR

Orthodontically induced external apical root resorption (OEARR) is a well-known adverse effect of tooth movement and is largely driven by the local sterile inflammation and cellular responses in the periodontal ligament (PDL). The severity of OEARR correlates strongly with the magnitude and duration of orthodontic force. In vivo studies in rat models have shown that excessive force leads to more odontoclasts, deeper resorption lacunae, and increased expression of inflammatory mediators in the apical region of the root [44]. These responses are particularly evident in areas of compressed PDL, where hypoxia and tissue damage initiate autophagy and apoptosis, followed by the recruitment of pro-inflammatory cells [9,10].

**Figure 3:**
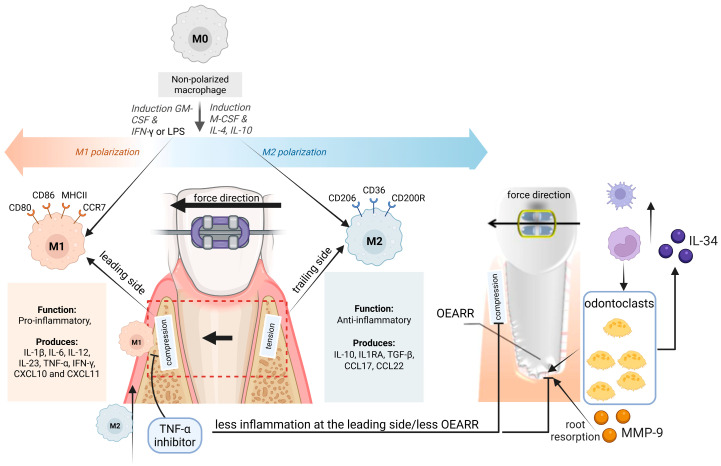
Polarized macrophage responses shape inflammation and root resorption during orthodontic tooth movement. Orthodontic force application triggers polarization of non-polarized macrophages (M0) into either M1 (pro-inflammatory) or M2 (anti-inflammatory) phenotypes. M1 polarization is induced by granulocyte-macrophage colony-stimulating factor (GM-CSF), interferon-γ (IFN-γ), or lipopolysaccharide (LPS), and is characterized by expression of surface markers CD80, CD86, MHCII, and CCR7. M1 macrophages localize predominantly at the leading (compression) side of the moving tooth and secrete pro-inflammatory cytokines including IL-1β, IL-6, IL-12, IL-23, TNF-α, IFN-γ, CXCL10, and CXCL11, which may exacerbate tissue inflammation and promote root resorption. Conversely, M2 polarization is induced by M-CSF, IL-4, and IL-10, and is characterized by expression of CD206, CD36, and CD200R. M2 macrophages accumulate at the trailing (tension) side, producing anti-inflammatory mediators such as IL-10, IL-1RA, TGF-β, CCL17, and CCL22, contributing to inflammation resolution. TNF-α inhibition can mitigate inflammation and reduce OEARR severity at the compression site. Moreover, compressive forces promote IL-34 expression, which stimulates odontoclast formation and upregulation of MMP-9, thereby contributing to root resorption. This polarized immune response modulates the extent of OEARR during orthodontic treatment. Created in BioRender. Steinberg, T. (2025) https://BioRender.com/nodkhxi.

A critical factor in this inflammatory cascade is macrophage polarization. Circulating monocytes infiltrating the PDL under compressive stress differentiate into M1 (pro-inflammatory) or M2 (anti-inflammatory) macrophages, depending on the local cytokine milieu. M1 macrophages, stimulated by interferon-gamma (IFN-γ) and granulocyte-macrophage colony-stimulating factor (GM-CSF), are predominantly found on the compression side and secrete cytokines such as TNF-α, IL-1β, and CXCL10, which amplify local inflammation and contribute to clastic cell activation [45]. In contrast, M2 macrophages are more abundant on the tension side and promote tissue repair via IL-10, IL-1RA, and TGF-β production. This polarization behavior and its link to OEARR are schematically summarized in Figure 3, which shows the differential roles of M1 and M2 macrophages, their signature cytokines, and their spatial distribution along the compression and tension sides of the periodontium during OTM. For a summary, see Table 2.

He et al. demonstrated in a mouse model that blocking TNF-α not only reduced M1 macrophage infiltration but also mitigated apical root resorption without impeding tooth movement [45]. This suggests that M1/M2 imbalance is not merely a marker but a direct contributor to OEARR. Further supporting this, Kohno et al. reported that IL-34, a cytokine involved in monocyte and macrophage lineage commitment, is highly expressed in odontoclasts during severe resorption events [46]. IL-34, together with macrophage colony-stimulating factor (M-CSF), promotes osteoclast and odontoclast differentiation and is linked to elevated expression of matrix metalloproteinase-9 (MMP-9), a key enzyme in collagen breakdown of the root cementum [46,47].

At the root surface, mechanical stress and inflammation compromise the cementoblast layer. Studies using OCCM-30 cementoblasts have shown that hypoxia downregulates HIF-1α, leading to caspase-dependent apoptosis or necrosis [10]. This disruption exposes the mineralized root matrix, enabling odontoclast adhesion and activity. These cells, derived from the monocyte/macrophage lineage, express high levels of MMP-9 and MMP-13, which degrade type I collagen in the cementum [47]. Their differentiation is regulated by the RANK/RANKL/OPG axis, which is shifted in favor of RANKL during OTM due to the influence of TNF-α and IL-1β secreted by M1 macrophages [48].

In clinical contexts, host genetic predisposition appears to influence OEARR severity. A systematic review by de Ávila Andrade et al. identified IL-1β and P2RX7 gene polymorphisms as consistently associated with increased risk of root resorption in orthodontic patients [49]. For example, Pereira et al. found that individuals with specific P2RX7 variants had significantly higher incidence of OEARR, suggesting that innate immune sensitivity to ATP may modulate inflammatory responses in the PDL [50].

Therapeutic strategies targeting these mechanisms are emerging. In animal models, TNF-α inhibition reduced root resorption lesions [45]. Another translational approach involves the use of RGD-mimetic peptides such as Cilengitide, which blocks integrin αvβ3–mediated adhesion of clastic cells to the root matrix, thereby impairing their resorptive function [51]. Together, these findings provide a mechanistic basis for interventions aimed at modulating macrophage polarization, cytokine signaling, and matrix degradation to prevent OEARR. The importance of these molecules for OTM is described in the following section.

Another possibility for the loss of root cementum, independent of inflammation-induced odontoclast activation and associated MMP-driven cementum matrix degradation, appears to be the presence or absence of HIF-1a. This possibility should be considered because in vitro studies on the immortalized cementoblast cell line OCCM-30 have shown that hypoxia reduces the expression of HIF-1α, which is regulated via MAP kinases ERK1/2, and that this reduction is causal (a) for the induction of caspase-driven (caspases: 3, 8 and 9) apoptosis or optionally (b) necrosis of the cementoblasts [10], as shown in Section 3.3.1).

**Executive Summary**

OTM triggers monocyte-derived macrophage polarization: M1 at the compression side promotes inflammation and root resorption; M2 at the tension side aids resolution. Manipulating the M1/M2 ratio (e.g., via TNF-α inhibition) offers a strategy to reduce OEARR severity.

**Table 2:** Macrophage polarization and impact on OEARR.

Macrophage Type	Inducers	Secreted Mediators	Function in OTM	References
M1	IFN-γ, GM-CSF, LPS	IL-1β, TNF-α, CXCL10/11	Pro-inflammatory; promotes OEARR	[45,52,53]
M2	IL-4, IL-10, M-CSF	IL-10, IL-1RA, CCL17/22	Anti-inflammatory; tissue repair	[45,53]
IL-34	Compressive force induced	Stimulates odontoclast formation	Linked to cementum resorption	[46,54]

#### 3.3.3. The RANK RANKL OPG System Orchestrates OTM-Related Bone and Root Resorption

**Figure 4:**
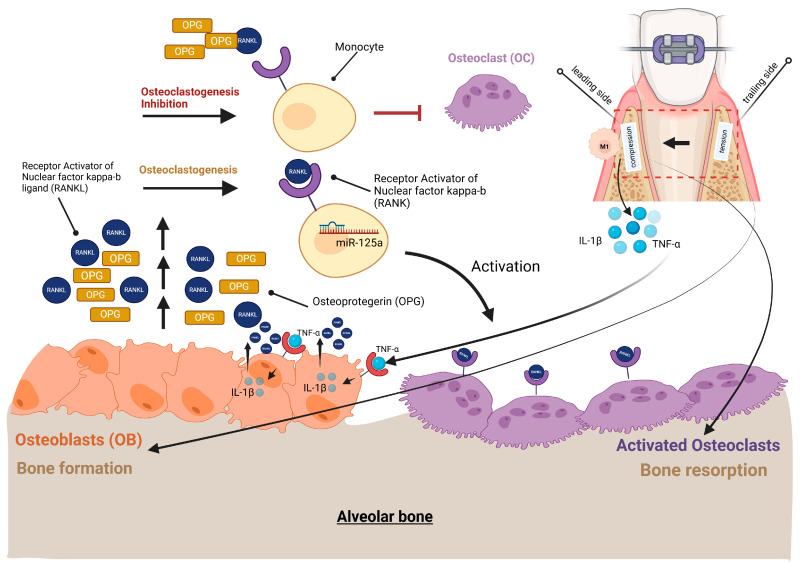
RANK/RANKL/OPG axis and cytokine-mediated regulation of osteoclastogenesis in orthodontic bone remodeling. Orthodontic force applied to the tooth initiates a cascade of cellular events within the periodontium, prominently involving the regulation of osteoclastogenesis via the RANK/RANKL/OPG axis. Osteoblasts (OBs) and other stromal cells produce receptor activator of nuclear factor kappa-B ligand (RANKL), which binds to its receptor RANK expressed on monocyte-derived osteoclast precursors, driving their differentiation into mature osteoclasts. Osteoprotegerin (OPG), a decoy receptor secreted by osteoblasts, inhibits this process by sequestering RANKL and preventing RANK activation. miR-125a is shown to positively regulate RANK expression, enhancing osteoclastogenesis. Inflammatory cytokines such as IL-1β and TNF-α, produced at the leading (compression) side of the tooth, further stimulate osteoclast differentiation and activation, enhancing bone resorption. In contrast, bone formation is maintained by osteoblasts at the trailing (tension) side. The RANK/RANKL/OPG balance and cytokine milieu collectively modulate alveolar bone remodeling during orthodontic tooth movement. Created in BioRender. Steinberg, T. (2025) https://BioRender.com/7nww9ep.

In addition to the OEARR described above, the pro-inflammatory presence of M1 macrophages on the leading side is partly responsible for the release of inflammatory mediators such as TNF-α and IL-1β with TNF-α favoring the expression of IL-1β (Figure 4). Further, TNF-α in osteoblasts induced the expression of RANKL via IL-1β, as demonstrated in IL-1 receptor 1 (IL1-RI)-deficient mice in vivo [48]. RANKL, for its part, is one of the central molecules for the maturation of cells that resorb hard tissues, such as the previously described odontoclasts, which resorb root cementum, and osteoclasts, which facilitate OTM via bone resorption [55] (Figure 4).

Osteoclasts can either arise under the influence of microRNAs (miRs), such as the already mentioned miR-125a, through the transition from monocytes to osteoclasts mediated by the transcription factor (TF) nuclear factor kappa B (NF-κB), or through the differentiation of macrophages into osteoclasts. In both cases, this requires the binding of the soluble ligand RANKL to its cellular receptor RANK and the associated activation of NF-κB [56]. In the case of the involvement of miR-125a, this miR suppresses the expression of monocyte-specific genes (e.g., tumor necrosis factor, alpha-induced protein 3 or A20 [TNFAIP3], insulin-like growth factor-1 receptor [IGF1R] and IL-15) in monocytes and thereby promotes the transition to osteoclasts (Figure 4). The expression of miR-125a is directly dependent on the activity of the TF NF-κB, as shown by NF-κB inhibition experiments on human primary monocytes in vitro [52].

Regardless of how odontoclasts or osteoclasts are formed, the expression of two molecules is an essential prerequisite for their formation and maturation. The first molecule is macrophage colony stimulating factor (M-CSF), which regulates the long-term survival and proliferation of monocytes. Other functions of M-CSF are to force the differentiation of bone marrow progenitor cells into osteoclast progenitor cells and to enhance the expression of RANK in bone marrow cells. These functions of M-CSF in osteoclastogenesis are extensively discussed in a review by Asagiri and Takanayagi [57] (Figure 4).

The second molecule is the cytokine RANKL which belongs to the TNF family and which controls the fusion and thus differentiation of mononuclear osteoclast precursor cells into multinucleated mature osteoclasts. In addition to RANKL, there is a second cytokine, the already mentioned inflammatory mediator TNF-α, which mediates osteoclastogenesis. In so-called canonical NF-κB signaling, both molecules induce the translocation of the NF-κB p65/p50 heterodimer into the cell nucleus through proteasomal degradation of the NF-κB inhibitor IκB-α [58]. The activity of the transcription factor (TF) NF-κB then leads to the expression of two further TFs, namely cellular FOS gene product (c-fos, is part of the AP-1 transcription complex) and nuclear factor of activated T cells c1 (NFATc1), whereby the primary function of NFATc1 in the early phase of osteoclstogenesis is to down-regulate the expression of RANKL signaling inhibitors like B-cell lymphoma 6 (Bcl6), since Bcl6 inhibits NFATc1 expression [59]. The activity of the TF c-fos leads to increased expression of NFTAc1, which induces the expression of osteoclast-specific biomarkers in later stages of osteoclastogenesis. Biomarkers inherent in NFATc1 responsive osteoclasts among others include tartrate resistant alkaline phosphatase (TRAP), the protease cathepsin K and MMP-9 [60], which has already been mentioned several times in the OTM context, since it can degrade the collagen matrix of bone and root cement alike. With regard to the TF NFATc1, recent studies on knockout mice have shown that of the existing isoforms, the NFATc1 short isoform, Nfatc1/αA, in particular is essential for osteoclastogenesis, since without it, fusion into the multinucleated mature osteoclasts cannot occur [61].

Since RANKL belongs to the TNF family, its cellular counterpart OPG is a member of the TNF receptor superfamily. OPG is a master regulator of bone homeostasis and protects the bone from drastic resorption. OPG as a secretory protein exerts its biological function after proteolytic cleavage of a signal peptide and consecutive dimerization. As a mature homodimer, it can then bind RANKL extracellularly and in this way prevents the signaling of RANK and RANKL just described, thereby abolishing the maturation process from pre-osteoclasts to osteoclasts. In this way, the molecular system consisting of RANK, RANKL and OPG controls the current bone supply, with a shift in the ratio in favor of RANKL leading to an underrepresentation of OPG and promoting bone degradation. Such an imbalance between RANKL and OPG with a reduced OPG presence is characteristic of osteoporosis and inflammatory diseases such as rheumatoid arthritis and periodontitis. But it is also the basis of bone loss during OTM, as tooth movement would not be possible without this imbalance (Figure 4).

Further studies of OPG on PDL cells cultured in vitro have shown that, similar to the transition from monocytes to osteoclasts in connection with the down-regulation of the expression of monocyte-specific genes (see above in this Section 3.3.3), the regulation of OPG expression may also be controlled via miRs. The authors came to this conclusion after subjecting PDL cells to tensile and compression forces. During compression forces, they detected a miR, namely miR-3198, which was significantly upregulated and were then able to show via mechanistic inhibition experiments with a miR-3198 inhibitor that miR-3198 drastically reduced the expression of OPG [62].

Another ligand of OPG is the TNF-related apoptosis-inducing ligand (TRAIL). Regarding TRAIL, its binding to OPG prevents TRAIL interaction with its death receptors (DRs), thereby preventing TRAIL-bound apoptosis. In this way, OPG can not only intervene in the regulation of apototic processes in the context of OTM, but also in the context of other biological processes, especially the development of cancer. It was shown here that OPG, for example, prevents the apoptosis of ovarian cancer cells mediated via TRAIL and does so with the involvement of the integrins αvβ3 and α5β5 [63]. In connection with the OTM, it was shown in animal experiments that increased expression of TRAIL could be detected on both the leading and trailing sides of the OTM [64]. For a summary, see Table 3.

**Executive Summary**

The RANK-RANKL-OPG axis governs osteoclastogenesis during OTM. A shift toward increased RANKL or reduced OPG promotes bone and root resorption. miRNAs (e.g., miR-125a, miR-3198) regulate this balance. Targeting this axis allows precise control over OTM and OEARR.

#### 3.3.4. Summary of the Cellular and Molecular Basis of OTM

In summary, the explanations on the cellular and molecular basis of OTM show that the prerequisite for this is a mechanical-induced, i.e., not bacterial-induced, inflammation, which occurs in a classic manner at the level of the mediators and the inflammatory cells involved. What is interesting is the increasing evidence in the last decade that even in the context of OTM, molecules that regulate osteoclast differentiation, such as monocyte-specific biomarkers or OPG, are themselves regulated in their expression by miRs. In the context of the previous explanations (i) the protease-driven hyalinization as an obstructive factor of the OTM should be emphasized and (ii) the fact that the direction of OTM creates a trailing side (tension) and a leading side (compression) that drive bone remodeling. It should also be emphasized that (iii) in the course of OTM, external root resorption occurs due to inflammation, which is an extremely unpleasant side effect. These three aspects, which are discussed in detail in a recently published article by Zheng and co-workers [44], form the basis for the following suggestions on translational applications and perspectives to help shape orthodontic tooth movement in the desired way in the future at the cellular and molecular level.

### 3.4. Approaches for Shaping Orthodontics of the Future Concerning Gene Polymorphisms as Well as Concepts Based on Cells, Biomaterials and Molecules and Small Molecules, Respectively

As can be seen from the explanations on the cellular and molecular basis of OTM, hyalinization and OEARR are the most significant undesirable effects in the tooth movement context. This is because hyalinization impedes tooth movement, as it prevents the sprouting of PDL-regenerating macrophages and fibroblasts within the lag or arrest phase (phase terminology depends on the respective model of OTM; see Section 3.2). The danger with OEARR is that it may be potentially irreversible if it is too pronounced and thus implies loss of root structure [65], which may cause tooth loss. What both undesirable effects have in common is that their degree of severity is obviously dependent on the amount of force applied during treatment (for hyalinization, see Ren et al. [66]; for hyalinization, see Jamali et al. [67]; for OEARR, see Zhong et al. [68]), and thus related to the extent of the force-induced inflammatory reaction within the periodontium. For the OEARR, their link to orthodontic force magnitude and inflammatory responses is explored in a review article that focuses on the ambivalence of the inflammatory response in the context of OTM [69]. Hence, in relation to OEARR, a paradox is emerging with a focus on the inflammatory reaction, as inflammation determines the speed of tooth movement on the one hand, but can also be destructive for the root and thus the tooth if excessive and uncontrolled [70]. Against this background, inflammation management seemingly plays a very important role for the dentist when planning and performing OTM.

#### 3.4.1. The Role of Gene Polymorphisms in Inflammation per Se and Inflammation-Related OEARR

As described in Section 3.3.3, the resorption of hard substances, i.e., root cementum and bone, is dependent on the maturation of odontoclasts and osteoclasts mediated by inflammatory mediators including TNF-α, IL-1β and RANKL. Among others, a central mediator here is obviously IL-1β, which is one of the most intensively researched in the context of inflammation per se and inflammatory periodontopathies. With regard to inflammation and a connection with IL-1, a patient study from 2002 showed that four polymorphisms in IL genes (including IL-1α/β), which have been shown to modulate inflammation, correlated with significantly higher plasma levels of C-reactive protein (CRP) [71], a veritable biomarker of systemic inflammation. For the reader who wants to learn more about the role of CRP as a marker for systemic inflammation, the review by Amezcua-Castillo et al. appears interesting, as it deals very intensively with CRP in the context of coronary heart disease [72]. In addition, previous studies have shown that certain polymorphisms in the IL-1α gene yielded increased IL-1α protein levels in the GCF of periodontitis patients [73]. For the interested reader, the connection between IL-1polymorphisms (applies to both IL-1α and IL-1β) and susceptibility to disease is extensively discussed in a review by Khazhim and colleagues [74]. With respect to periodontopathies, detailed studies on polymorphisms of various interleukins have shown that as an example, IL-4 and IL-13 polymorphisms do not play a role in inflammation-related periodontitis [75], but that in addition to IL-1α IL-1β, they do have a role to play [76].

It therefore seems obvious that gene polymorphisms for the IL-1 gene have also been investigated in connection with OEARR in order to clarify a genetic predisposition in this regard. In a recent review article on OEARR and its association with possible gene polymorphisms, numerous genes in the context of IL-1 and bone resorption, including RANKL, were examined. From their investigation, the authors concluded that almost all of the 21 studies examined suggested an association of OEARR with polymorphisms in the IL-1 gene, while most of the polymorphisms of the other genes examined, among others the IL-1 receptor antagonist (IL-1RN, see below), only showed an association in isolated studies. Furthermore, the authors were able to show that a polymorphism in the purinergic-receptor-P2X ligand-gated ion channel 7 (P2RX7 is a purinergic receptor which, inter alia, serves as a pattern recognition receptor for extracellular ATP) gene is obviously causally involved in the etiopathogenesis of OEARR [52]. This causality is based, for example, on a retrospective patient study with a total of 195 patients, which was able to identify a P2RX7 gene polymorphism as a possible factor of susceptibility to EARR on the basis of a multiple-linear regression model [50]. Concerning IL-1, there are polymorphisms for both IL-1α and IL-1β. While studies on patients did not reveal any relevance of the polymorphisms investigated for IL-1α for the OEARR [77]), such relevance was found for IL-1β and IL-1β in conjunction with IL1RN [78].

However, in connection with clinical studies and the search for correlations between gene polymorphisms of suspected target genes and periodontal disease, it should be noted that the number of study participants, their age and gender must be taken into account when assessing whether a polymorphism is relevant or not. Together with the parameters for assessing root resorption per se, e.g., based on imaging procedures, the aforementioned influencing variables can be decisive for the significance of a respective gene polymorphism.

In addition to the tendency to develop root resorption, gene polymorphisms may also be used in the future to predict whether a patient is more likely to require a short or long treatment period in the context of indication-specific orthodontic treatment. This perspective is based on a Korean study of patients who underwent orthodontic treatment following premolar extraction. After collecting and analyzing the clinical follow-up data, the study designers divided the patients into extremely short and extremely long treatment time groups based on the average treatment time. The authors were able to determine a total of nine polymorphisms in six genes from saliva samples, which were either specific for patients with extremely short or extremely long treatment periods [79]. For a summary, see Table 4.

**Executive Summary**

Gene polymorphisms, especially in IL-1 and P2RX7, affect individual inflammatory responses and susceptibility to orthodontically induced external apical root resorption (OEARR). Genotyping may aid risk prediction and personalization of orthodontic protocols.

#### 3.4.2. Conclusion on Gene Polymorphisms and Future Perspectives in the Context of Orthodontic Treatment and OTM

With inflammation-associated hyalinization and OEARR, orthodontics today still faces two major challenges that will shape the future, despite all the diagnostic and therapeutic findings. With the gene polymorphisms shown in Section 3.4.1 in the context of OEARR, polymorphisms affecting the IL-1 gene and the P2RX7 gene have emerged in a causal relationship with OEARR according to current knowledge. In addition, there is growing evidence that gene polymorphisms may also be used in the future to predict treatment times for certain orthodontic indications. Against this background, a possible recommendation for the design of orthodontic treatment in the future may be to screen a patient for candidate gene polymorphisms prior to treatment, which are related to (i) a tendency to inflammation per se, (ii) the risk and degree of OEARR and (ii) the prediction of treatment time. In the future, such a pre-therapeutic screening may not only provide the practitioner with information about the patient’s status with regard to the points (i) to (iii) just mentioned, but also give him a clue how to organize the treatment forces initially and in the course of the respective treatment cycle in order to achieve his therapeutic goal as quickly as possible and with as few side effects as possible.

#### 3.4.3. Strategies for PDL Regeneration as a Result of Hyalinization

Building upon the molecular mechanisms detailed in Section 3.3, the following strategies aim to accelerate the resolution of hyalinization and promote timely regeneration of the periodontal ligament (PDL). Force-induced compression of the PDL at the leading side, especially in the initial phase of OTM, causes not only cell death but also protease-driven destruction of ECM, particularly collagen (see hyalinization: Introduction and Section 3.3 in this regenerative process, Section 3.3.1). However, since tooth movement can only continue once the hyalinization in the PDL has been eliminated, the regeneration of the PDL, with a preference on the reconstitution of the bioinactive collagen, is of primary importance. As described in Section 3.2, the lag phase of orthodontic tooth movement (OTM) is characterized by a distinct inflammatory response that is integral to the process of tooth realignment. Central to this phase is the activation of MMPs, notably MMP-1, MMP-8, and MMP-13, which mediate the degradation of the ECM in the PDL, primarily affecting collagen fibers [13,38,80]. This proteolytic activity culminates in the formation of a hyalinized zone, a hallmark of the lag phase, where the PDL matrix is significantly disrupted, hindering the subsequent regenerative processes required for continued tooth movement. The persistence of this hyalinization is a critical determinant of the duration of the lag phase, which can last several weeks, until macrophages and other phagocytic cells remove the degraded ECM components [81]. To accelerate the resolution of this phase and mitigate its inhibitory effects on OTM, strategies that enhance the rapid removal of collagen debris have been explored. One promising approach involves the enhancement of phagocytosis by M2 macrophages, which can be potentiated through the administration of IFN-γ. This cytokine has been shown to act as a potent macrophage activator, potentially facilitating the swift clearance of the hyalinized matrix and allowing for a quicker transition to the next phase of tooth movement [82].

The regeneration of the PDL matrix, following its destruction in the lag phase, is essential for the resumption of tooth movement. One innovative approach to enhance matrix regeneration involves the injection of plant-based recombinant human collagen (rhCol1), a highly biocompatible material that can be proteolytically tailored to achieve desired fragment sizes, thus mimicking the structural properties of native collagen fibers. This method, which has already seen clinical success in periodontitis therapy, offers a minimally invasive option for regenerating the hyalinized PDL matrix during OTM. Furthermore, the use of collagen-based carrier materials, such as hydrogels, facilitates the spatio-temporal delivery of cytokines and growth factors like for instance IFN-γ or bone morphogenetic proteins (BMPs), to accelerate PDL regeneration and promote bone formation, as reported in recent studies [83,84].

An additional promising strategy for modulating the hyalinization process involves the use of MMP inhibitors. In the field of periodontology, the FDA-approved MMP inhibitor Periostat, a low-dose doxycycline formulation, has been employed to prevent the proteolytic breakdown of the periodontal ECM. This antibiotic, known for its ability to chelate calcium and zinc ions, impedes the activity of a broad range of MMPs, thereby reducing ECM degradation [85]. Recent advancements in MMP inhibition have led to the development of more selective MMP inhibitors, such as andecaliximab, a monoclonal antibody targeting MMP-9, which has been already applied in clinical trials to defeat chronic inflammatory diseases like for instance rheumatoid arthritis [86]. MMP-9 plays a pivotal role in osteoclast-mediated bone resorption and is involved in the degradation of both PDL matrix and bone matrix (COL1 and osteopontin [OPN]), suggesting that its inhibition could mitigate the adverse effects of OTM-induced bone resorption [87,88].

Another avenue for enhancing OTM involves the modulation of blood flow to the periodontium, which has been shown to influence the inflammatory response and cellular activities within the PDL. Recent animal studies suggest that the administration of arginine, an amino acid involved in nitric oxide (NO) synthesis, can accelerate tooth movement by promoting vasodilation and enhancing blood flow to the periodontium. The concomitant administration of citrulline, which is enzymatically converted to arginine, further enhances NO production, thereby facilitating macrophage extravasation and potentially accelerating the reversal of hyalinization [89].

As the hyalinized matrix is cleared, the regeneration of the PDL requires the migration of fibroblasts, which are responsible for synthesizing new ECM components, particularly collagen. Periostin, an ECM protein known to mediate fibroblast migration and adhesion, has shown promise in enhancing the regeneration of the PDL during OTM. It facilitates the conversion of fibroblast precursor cells into myofibroblasts, which are essential for wound healing and ECM deposition [90]. The combination of periostin with connective tissue growth factor (CCN2), a matrix-associated protein that enhances fibroblast attachment and proliferation, may further accelerate PDL regeneration. This synergistic approach has yielded positive results in animal models of wound healing and suggests a potential strategy for improving the outcomes of OTM [91,92]. Additionally, periostin may serve as a valuable diagnostic marker for monitoring the severity of inflammation during OTM. Its presence in sulcus fluid correlates with the extent of the inflammatory response and may provide a non-invasive means of assessing treatment progress in orthodontic patients [93]. For a summary, see Table 5.

**Executive Summary**

PDL regeneration following hyalinization is critical for resuming tooth movement. Strategies include recombinant collagen (rhCol1), cytokine-loaded hydrogels, and MMP inhibition. Blood flow modulators and periostin–CCN2 axis also promote ECM repair and fibroblast activation.

#### 3.4.4. Summary Highlighting Key Strategies to Overcome Hyalinization

As previously discussed in Section 3.3, the early phase of OTM involves ECM degradation and cell death. This section summarizes therapeutic strategies designed to overcome the resulting hyalinization and support PDL repair. This degradation, resulting in hyalinization, hinders further tooth movement until the matrix is cleared by macrophages and other phagocytes. Strategies to accelerate hyalinization resolution include enhancing macrophage activity via IFN-γ, as well as promoting PDL regeneration using plant-based rhCol1 and collagen-based hydrogels to deliver growth factors. Inhibitors of MMPs, such as doxycycline (Periostat) and more selective MMP-9 inhibitors, also present promising interventions to reduce ECM degradation and prevent excessive bone resorption. Additionally, modulating blood flow with arginine and citrulline to improve vasodilation has been shown to facilitate macrophage extravasation and accelerate the reversal of hyalinization. Further, periostin, a key protein in fibroblast migration, in combination with CCN2, may further enhance PDL regeneration and improve treatment outcomes. These approaches, while still under investigation, offer promising avenues to optimize OTM and reduce unwanted side effects, such as external root resorption.

#### 3.4.5. Biomaterial Concepts for Spatio-Temporally Directed Release of RANKL and Osteoclasts from Inducible Pluripotent Stem Cells (iPCs) via Injectable Hydrogels

Informed by the role of osteoclastogenesis in bone remodeling outlined earlier, biomaterial-based methods such as hydrogel delivery are being developed to spatially control osteoclast activity. OTM remains a complex process involving numerous cellular and molecular interactions, including osteoclastogenesis, which is crucial for bone resorption. However, the challenge remains in overcoming the inhibitory effects of hyalinization, which can impede tooth movement. As recent studies have highlighted, the localized delivery of biologically active molecules, such as the RANKL, has shown promise in accelerating OTM by promoting osteoclast differentiation [96]. RANKL is a central regulator of osteoclastogenesis, activating the RANK receptor on osteoclast precursors and triggering downstream signaling cascades, including NF-κB, which promote osteoclast differentiation and activity.

Incorporating RANKL within biomaterial delivery systems like injectable hydrogels provides a spatio-temporally controlled release platform that enhances the efficacy and safety of osteoclastogenesis during orthodontic tooth movement (OTM). Hydrogels can be broadly categorized as natural (e.g., gelatin, alginate, collagen, chitosan) and synthetic (e.g., polyethylene glycol [PEG], poly(lactic-co-glycolic acid) [PLGA]), each offering distinct physicochemical properties relevant to drug delivery. Natural hydrogels are typically biocompatible and biodegradable through enzymatic pathways and exhibit relatively fast degradation rates (days to a few weeks), which may be beneficial for short-term, high-burst release of bioactive molecules such as RANKL. In contrast, synthetic hydrogels offer precise control over crosslinking density and degradation kinetics, ranging from weeks to several months, allowing sustained and programmable release depending on the polymer backbone and crosslinker used [96,97]. For example, PEG-based hydrogels have demonstrated slow, predictable degradation ideal for long-term RANKL administration, while chitosan-based hydrogels allow for pH- and temperature-responsive release [97,98]. Such design flexibility is critical in tailoring RANKL delivery to different treatment phases or patient-specific needs. Future developments may involve hybrid or stimuli-responsive hydrogels that dynamically respond to orthodontic force levels, enabling “on-demand” release of osteoclastogenic factors. Additionally, such systems could be used to deliver osteoclast precursors or cytokines that further modulate bone remodeling at the site of OTM. By combining the regenerative properties of stem cells such as iPC-derived osteoclasts with these advanced biomaterials, it becomes feasible to create a dynamic, controllable microenvironment that accelerates bone resorption while minimizing adverse effects. Thus, a promising therapeutic strategy to enhance OTM involves the differentiation of inducible pluripotent stem cells (iPCs) into osteoclasts, followed by their incorporation into injectable hydrogels. iPCs are a valuable source of osteoclast precursors, offering a unique advantage due to their ability to be derived non-invasively from various tissues such as urine [99]. This allows for the creation of personalized therapeutic strategies without the need for invasive procedures. Recent protocols for osteoclast differentiation typically involve the use of vitamin D3 to stimulate RANKL expression, and transforming growth factor (TGF)-β to promote RANK and NF-κB synthesis, key pathways in osteoclastogenesis [100]. Once differentiated, osteoclasts can be loaded into hydrogels that have been optimized for sustained release and local delivery to the site of tooth movement. The ability to precisely control the differentiation of iPCs into osteoclasts and their delivery to specific sites offers exciting opportunities for enhancing the efficiency and predictability of OTM. This approach is poised to address limitations in current orthodontic treatment by allowing more localized and efficient bone resorption, reducing the risk of adverse side effects such as root resorption or uncontrolled inflammation. For a summary, see Table 6.

**Executive Summary**

Injectable hydrogels enable localized delivery of RANKL and iPC-derived osteoclasts, promoting targeted bone remodeling. This method allows precise temporal control of OTM while minimizing systemic effects.

#### 3.4.6. Bone Remodeling, Osteoclastogenesis, Root Resorption and Its Prevention in OTM

Bone remodeling plays a pivotal role in orthodontic therapy, as it is essential for tooth movement and maintaining root integrity. Osteoclasts, which resorb bone, are key players in this process, while osteoblasts are responsible for bone formation. In the context of OTM, osteoclast activity on the leading side of the tooth allows for the necessary bone resorption that facilitates tooth movement, while osteoblasts regenerate bone on the trailing side. However, an imbalance in this process can lead to undesirable effects such as root resorption, a common and concerning complication of orthodontic treatment [44]. Emerging strategies focus on modulating the differentiation of stem cells, such as those derived from urine or the PDL, to guide the process of bone resorption and repair. Co-culturing urine-derived stem cells with PDL stem cells has demonstrated the potential to direct these cells toward cementogenic differentiation, a process that may prevent or reverse root resorption [103]. A further promising approach to address root cementum regeneration based on a biomimetic cementum, comprising a combination of bioskiving and fluorine-containing biomineralization [104].

Integrating Arg-Gly-Asp (RGD) sequence mimetics like Cilengitide has emerged as a promising strategy to inhibit the adhesion of osteoclasts and odontoclasts to the extracellular matrix (ECM) of alveolar bone and root cementum. These cells utilize integrins to bind to the ECM, facilitating bone resorption. Cilengitide, a cyclic peptide that mimics the RGD sequence, competitively inhibits integrin binding, thereby reducing osteoclast and odontoclast adhesion and activity. Recent studies have demonstrated that Cilengitide effectively diminishes osteoclast-mediated bone resorption in vitro and in vivo, suggesting its potential in managing bone resorption-related conditions. For instance, a study by Guo et al. [51] reported that αvβ3 integrin inhibitor Cilengitide treatment significantly decreased osteoclast differentiation and activity, leading to reduced bone resorption in a mouse model [105,106]. As extensively reviewed by Patel et al., 2022, root resorbing odontoclasts are very similar to osteoclasts (see also Section 3.3.2), it appears likely that Cilengitide hampers their activity by preventing adhesion to the RGD sequence of root-related ECM [107]. These findings underscore the therapeutic potential of RGD mimetics like Cilengitide in controlling osteoclast and odontoclast adhesion, thereby preserving alveolar bone and root cementum integrity.

Another promising therapeutic avenue involves controlling inflammation, a key driver of osteoclastogenesis. Since inflammatory responses in OTM are inherently osteoclastogenic, modulating the inflammatory response at the molecular level can help prevent excessive bone resorption and root damage [108,109]. For a summary, see Table 7.

**Executive Summary**

Innovative approaches to prevent OEARR include ECM-integrin inhibitors (e.g., Cilengitide), biomimetic cementum, and inflammation modulation. These aim to preserve root structure during force-driven resorption.

#### 3.4.7. PROTACs: Targeted Protein Degradation for Controlling OTM

Proteolysis-targeting chimeras (PROTACs) represent a groundbreaking class of small molecules that are capable of selectively targeting and degrading proteins of interest (POIs) within cells, leveraging the body’s natural ubiquitin–proteasome system. PROTACs consist of a bifunctional molecule with a ligand for the POI on one end and a ligand for an E3 ubiquitin ligase on the other end, facilitating the ubiquitination and degradation of the POI [110]. This innovative approach offers several advantages over traditional inhibition strategies, such as small molecule inhibitors or monoclonal antibodies, by eliminating the target protein entirely rather than merely blocking its activity.

The use of PROTACs in the context of OTM is highly promising, particularly for modulating inflammatory signaling pathways that influence osteoclastogenesis. NF-κB, a TF central to the regulation of RANKL expression, plays a pivotal role in the osteoclast differentiation process. By targeting NF-κB with PROTACs, researchers can selectively degrade this TF, thus disrupting the inflammatory signaling that drives RANKL expression in osteoblasts and preosteoclasts [111,112]. This offers the potential to modulate osteoclastogenesis with high precision, reducing unwanted bone resorption and improving the overall effectiveness of OTM.

Additionally, the uptake mechanisms of PROTACs are a critical aspect of their function. These molecules typically enter cells Via endocytosis, often utilizing receptors such as folate receptors (through folate-coupling, [113]) or HER2 (through antibody coupling, [114]). This enables the targeted delivery of PROTACs to specific cells, enhancing their selectivity and minimizing off-target effects. Once inside the cell, PROTACs bind to the E3 ligase complex and the POI, leading to ubiquitination and proteasomal degradation. In the context of OTM, the targeted degradation of inflammatory mediators like NF-κB could potentially offer a new level of control over the bone remodeling process, allowing for precise manipulation of osteoclast activity and thus improving the outcomes of orthodontic treatment. For a summary, see Table 8.

**Executive Summary**

PROTACs offer targeted degradation of intracellular proteins (e.g., NF-κB), enabling fine-tuned inflammatory control during OTM. Their selective action holds potential to prevent excessive osteoclast activation and OEARR.

#### 3.4.8. Biologics: Infliximab and Caspase-1 Inhibition

Biologics such as infliximab, a monoclonal antibody that targets and neutralizes TNF-α, represent a highly targeted therapeutic option for managing the inflammatory components of OTM. By blocking TNF-α, infliximab prevents the downstream activation of NF-κB, a key mediator in osteoclastogenesis, as well as RANKL expression in osteoblasts [115,116]. The role of TNF-α in regulating osteoclast differentiation highlights its potential as a target for modulating OTM-associated bone resorption.

Another molecular strategy involves the inhibition of caspase-1, a protease responsible for the activation of pro-inflammatory cytokines like IL-1β. Caspase-1 has been shown to be upregulated during OTM, and its inhibition could reduce the osteoclastogenic response, potentially limiting root resorption and accelerating tooth movement without excessive bone loss [117,118]. In this context, both biologics targeting TNF-α and caspase-1 could offer complementary approaches to modulate the inflammatory response and improve OTM outcomes. For a summary, see Table 9.

#### 3.4.9. Topical Administration of JAK Inhibitors and Further Approaches in Controlling Inflammation During OTM

Inflammatory responses play a central role in the regulation of OTM, influencing both the pace and outcome of bone remodeling. Inflammation-triggered signaling pathways, particularly those mediated by interleukins and interferons, can drive osteoclastogenesis and hinder bone formation. A promising approach to modulate these inflammatory pathways involves the topical administration of Janus kinase (JAK) inhibitors, such as tofacitinib, which specifically targets the JAK/signal transducer and activator of transcription (STAT) signaling cascade. By inhibiting JAK, tofacitinib prevents the translocation of the STAT TF into the nucleus, thereby attenuating the inflammatory response mediated by cytokines [119]. JAK signaling is essential for the differentiation of M1 macrophages [120], a pro-inflammatory subset that promotes NF-κB activation and subsequent cytokine production, ultimately driving osteoclastogenesis [121]. Tofacitinib, already used topically for conditions like atopic dermatitis, has shown potential for localized inhibition of inflammatory pathways in OTM, reducing inflammation at the treatment site and promoting a more controlled and efficient tooth movement process.

Beyond JAK inhibitors, targeting specific cytokine receptors also offers an effective means to control inflammation during OTM. For instance, the use of IL-1 receptor (IL-1R) antagonists like anakinra and IL-6 receptor (IL-6R) antagonists like tocilizumab has gained attention in clinical settings for managing autoimmune diseases such as rheumatoid arthritis, as well as in mitigating the inflammatory response in conditions like corona virus disease (COVID-19) [122]. These antagonists prevent the binding of pro-inflammatory cytokines like IL-1α/β and IL-6 to their respective receptors, thus inhibiting downstream signaling that triggers secondary inflammatory mediators, including prostaglandins, cytokines, and chemokines [123]. By targeting these pathways, these antagonists reduce RANKL expression and may consequently also modulate osteoclastogenesis during OTM.

In addition to broader inflammatory control, more localized strategies targeting the osteoclast or odontoclast may offer a refined approach to regulating OTM. One such promising strategy involves the use of hydrogels or nanoparticle-based delivery systems to localize the application of anti-inflammatory cytokines such as IL-10. IL-10 has been shown to inhibit osteoclastogenesis by reducing RANKL expression and preventing the maturation of osteoclast and odontoclast progenitors [124]. Additionally, IL-10 impedes the activation of NFATc1, a key TF for osteoclast differentiation, by suppressing the expression of osteoclast-specific markers such as TRAP, cathepsin K, and integrin (αv)β3 [125,126]. By strategically applying IL-10 to the site of OTM, this approach could precisely modulate osteoclast maturation, reducing unwanted bone resorption while facilitating effective tooth movement.

Another innovative strategy for targeting osteoclasts and odontoclasts during OTM involves the use of monoclonal antibodies such as denosumab, which prevents the RANKL–RANK interaction. Denosumab, already approved for the treatment of osteoporosis, can block RANKL from binding to its receptor on osteoclast precursors, thus inhibiting their differentiation and activity [127]. This antibody-based approach offers a direct mechanism to control osteoclast and odontoclast function, providing a targeted intervention that can mitigate bone resorption during orthodontic treatment.

Taken together, the topical application of JAK inhibitors and cytokine receptor antagonists, alongside more localized approaches using IL-10 or monoclonal antibodies, represents a novel strategy for controlling inflammation and regulating osteoclast activity during OTM. These approaches offer significant potential for enhancing the precision, efficiency, and safety of orthodontic treatments, promoting more predictable outcomes and reducing adverse side effects like root resorption. For a summary, see Table 9.

#### 3.4.10. Semaphorin 3A: A Dual Regulator of Osteoblast Maturation and Osteoclast Inhibition in OTM

Semaphorin 3A (SEMA3A), a member of the semaphorin family of proteins, holds significant promise as a therapeutic agent in OTM. Semaphorins are secreted and transmembrane proteins that modulate cell migration and tissue remodeling, with SEMA3A playing a pivotal role in regulating bone formation and resorption. While still in early experimental stages, its potential as a candidate to stimulate bone formation on the traction side of OTM is emerging. Recent studies have demonstrated that SEMA3A, synthesized by osteoblasts in response to IL-1β signaling, inhibits osteoclast migration, thereby reducing bone resorption and promoting bone mineralization [128]. These findings suggest that SEMA3A may function as a key modulator in balancing bone resorption and formation during OTM.

In addition to its osteoclast-inhibiting effects, SEMA3A has been shown to influence osteoblast maturation. Specifically, in periodontal ligament (PDL) cells, SEMA3A binds to the PlexinA receptor, which activates C3 botulinum toxin substrate 1 (Rac1, a member of the Rac family of guanosine triphosphate phosphohydrolases [GTPases]) signaling. This, in turn, leads to the nuclear translocation of β-catenin, a critical effector that regulates osteoblast differentiation through the T-cell factor (TCF)/lymphoid enhancer-binding factor (LEF) TFs and the activation of runt-related TF (RUNX)2 and the zink finger TF OSX, the master regulators of osteogenesis [129]. The osteogenic potential of SEMA3A was further corroborated in recent OTM animal models, where SEMA3A, produced by trigeminal neurons during tooth movement, stimulated RUNX2 and OSX expression in PDL cells, promoting osteoblastic differentiation and mineralization [130].

Given its regulatory effects on both osteoblast and osteoclast activity, SEMA3A represents an attractive target for therapeutic interventions aimed at optimizing bone remodeling during OTM. The spatio-temporal control of SEMA3A delivery could be achieved through advanced biomaterial systems, such as injectable hydrogels, nanoparticles, or liposomes, which would allow for precise modulation of its effects at the desired site of action. Such technologies could enable a more controlled and efficient application of SEMA3A, enhancing bone formation on the trailing side of OTM while mitigating the risks of excessive bone resorption.

Overall, while SEMA3A’s role in OTM remains an area of active investigation, the growing body of evidence points to its potential as a transformative therapeutic approach for improving orthodontic outcomes by enhancing osteoblast maturation and inhibiting osteoclastogenesis [56,128]. For a summary, see Table 9.

**Executive Summary**

Monoclonal antibodies, JAK inhibitors, and cytokine antagonists are being explored to mitigate OTM-related inflammation. Localized delivery systems (e.g., hydrogels with IL-10) improve safety while retaining efficacy. These agents prevent OEARR by dampening osteoclastogenesis.

**Table 9:** Anti-inflammatory biologics and small molecules for OTM control.

Agent	Target	Effect	Delivery Mode	References
Infliximab	TNF-α	Reduces RANKL, suppresses NF-κB	Systemic/injectable	[115,116]
Anakinra	IL-1R	Blocks IL-1β signaling	Local/systemic	[122,123]
Tofacitinib	JAK1/3	Inhibits STAT phosphorylation	Topical/gel	[119,120]
Denosumab	RANKL	Prevents osteoclast maturation	Injectable	[127]
IL-10	Anti-inflammatory cytokine	Inhibits NFATc1, reduces TRAP/cathepsin K	Hydrogel/nanoparticle	[51,124,125]

#### 3.4.11. The Role of Artificial Intelligence (AI) Exemplified by Cancer Research and OTM

Artificial intelligence (AI), a transformative technology that increasingly permeates both daily life and biomedical science, holds immense promise for the future of healthcare. AI operates on two foundational pillars: machine learning (ML) and neural networks. Machine learning encompasses a broad spectrum of algorithms that detect patterns within large datasets, enabling the creation of predictive models that can inform decision-making across multiple domains. Neural networks, a specific subset of ML, function by mimicking the way the human brain processes information through interconnected nodes, allowing systems to learn and make complex predictions. As these technologies continue to advance, their applications across medical disciplines are growing exponentially.

*In cancer research*, AI is playing an increasingly pivotal role in precision medicine, particularly in the identification of “master genes” or critical molecular targets for specific cancers, such as breast and prostate cancer. For instance, recent studies have leveraged AI-based algorithms to analyze vast amounts of genetic data to uncover genes that drive cancer progression [131,132]. By utilizing machine learning models to recognize complex data patterns, AI can help identify biomarkers that are critical in the early detection of cancer and predict patient outcomes with unprecedented accuracy. Furthermore, AI has been used to design novel therapeutics by predicting how active substances interact with these master genes, creating a new avenue for drug development. Once identified, these drugs undergo rigorous testing for efficacy and tolerability, significantly accelerating the process of bringing targeted therapies to the clinic. The AI-driven approach can dramatically reduce the time and cost associated with traditional drug discovery methods [133,134,135,136].

This shift towards AI in cancer research exemplifies the capacity of computational tools to not only reveal new biological insights but also to guide therapeutic development with a level of specificity and efficiency that was previously unimaginable. Given the scope and complexity of genetic data in cancer biology, AI offers an invaluable tool for uncovering insights from otherwise intractable datasets. This concept of precision medicine, driven by AI, is not confined to oncology, as it has implications for many other areas of biomedical science, including orthodontics.

*In orthodontics*, the integration of artificial intelligence (AI) into orthodontics has advanced rapidly from conceptual applications to validated clinical tools. However, a distinction must be made between technologies already in use and those still under investigation. Table 10 provides a comparative overview of selected AI strategies, categorizing them as either validated clinical tools or experimental algorithms under development. This classification helps clarify the translational landscape and highlights both the current utility and future promise of AI in orthodontics.

Artificial intelligence (AI) is rapidly reshaping orthodontics by supporting diagnostic decision-making, predicting treatment outcomes, and enhancing the efficiency of care. AI models—especially deep learning algorithms—can process large imaging datasets and clinical records to extract diagnostic insights that rival or even exceed human performance. A recent systematic review and meta-analysis evaluated the diagnostic accuracy of AI models in orthodontic planning and concluded that AI significantly improves the precision of cephalometric landmark identification and automatic tooth segmentation compared to conventional methods [146,147]. These algorithms consistently achieved lower error margins and higher consistency than human raters, particularly in 2D and 3D imaging analyses.

In practical applications, AI systems have demonstrated excellent agreement with orthodontists in diagnostic classification. For instance, Bor et al. developed a clinical AI tool that classified Angle’s malocclusion with >90% agreement compared to expert orthodontists (κ = 0.90–0.95) [148]. Convolutional neural network (CNN)-based models also accurately predict treatment needs, such as extraction decisions, based on intraoral photographs. A study by Chang et al. trained a VGG19-based CNN using over 3000 clinical images and achieved a 92% classification accuracy (AUC = 0.96) in predicting the need for extraction in moderate to severe crowding cases [149]. These AI models also showed strong performance in detecting occlusal anomalies and skeletal discrepancies.

Another significant advancement involves AI-assisted cephalometric analysis. AI platforms now reliably identify landmarks and calculate skeletal parameters in lateral cephalograms with sub-millimeter precision. This accuracy has been confirmed in comparative studies, where automated systems matched or outperformed manual tracing in both speed and consistency [146]. In 3D diagnostics, AI-driven segmentation of CBCT data allows for accurate reconstruction of root and crown morphology, supporting real-time visualization of tooth movement and root resorption risk.

Despite these achievements, AI models are still limited by data generalizability and clinical integration challenges. Most systems are trained on retrospective datasets with limited diversity, and real-time clinical validation remains sparse. For example, while Bor et al.’s model performed well in malocclusion classification, its performance on continuous measurements such as Bolton ratios or root angulations was less accurate [148]. Moreover, “black-box” models limit interpretability, and ethical concerns remain regarding patient data privacy and clinical accountability.

Nonetheless, the consensus across systematic reviews is that AI offers substantial benefits when used as a clinical decision support tool rather than a replacement for the orthodontist. As algorithms continue to evolve and integrate into practice management software, their ability to augment diagnostics, reduce errors, and improve patient-specific planning is expected to grow. Ultimately, combining AI precision with orthodontic expertise may lead to more efficient, personalized, and predictable treatments. For a summary, see Table 11.

**Executive Summary**

AI is emerging as a key enabler in orthodontics for predictive modeling, treatment planning, and real-time monitoring. Integrating radiographic, genetic, and phenotypic data allows individualized therapy with greater accuracy and efficiency.

#### 3.4.12. Summary of the Section 3.4.5, Section 3.4.6, Section 3.4.7, Section 3.4.8, Section 3.4.9, Section 3.4.10 and Section 3.4.11

OTM is a complex process involving bone remodeling, osteoclastogenesis, and inflammatory responses. Strategies such as the spatio-temporal release of RANKL and osteoclasts from iPCs through injectable hydrogels show promise in accelerating OTM while minimizing side effects like root resorption. Biomaterials like hydrogels enable precise delivery of osteoclasts and cytokines to optimize bone resorption. Additionally, targeted protein degradation via PROTACs and biologics, including infliximab and caspase-1 inhibitors, offer new ways to modulate inflammatory signaling and osteoclast differentiation during OTM. Other approaches, like the topical use of JAK inhibitors, IL-10, and monoclonal antibodies (e.g., denosumab), provide more controlled regulation of inflammation and osteoclast activity, further enhancing treatment efficacy. SEMA3A, which simultaneously inhibits osteoclastogenesis and promotes osteoblast maturation, offers an exciting therapeutic potential for balancing bone resorption and formation in OTM. Lastly, the role of AI in both cancer research and orthodontics is highlighted. AI-driven tools are transforming treatment planning by enabling precision predictions, optimizing therapeutic outcomes, and personalizing care. By leveraging AI’s capabilities, orthodontic practices can improve treatment predictability, reduce complications, and enhance overall patient satisfaction, paralleling advancements in other medical fields like oncology. This integrated approach to OTM is poised to revolutionize orthodontic treatments and outcomes.

#### 3.4.13. Open Research Questions in the Future of Orthodontic Treatment

Despite significant advancements in orthodontic treatments, numerous critical research questions remain that must be addressed to fully harness the potential of emerging technologies such as cellular therapies, biomaterials, molecular interventions, and AI in shaping the future of orthodontics.

Cellular Therapies and Gene Polymorphisms

Cellular approaches, particularly involving induced pluripotent stem cells (iPSCs), have demonstrated promise in differentiating into osteoclasts and osteoblasts for the regulation of bone remodeling during OTM. However, substantial challenges persist in refining protocols to optimize the differentiation efficiency and safety of these cells. Future research should focus on improving the reproducibility and scalability of iPSC-derived therapies for clinical application. Additionally, the long-term risks associated with iPSC-derived cells, such as uncontrolled bone remodeling or tumorigenesis, need to be thoroughly investigated to ensure their safe use in clinical treatments.

Another crucial avenue of exploration is the role of gene polymorphisms, particularly those involving interleukins (IL-1α/β), in influencing inflammation and OEARR during orthodontic treatments. Understanding how genetic predispositions impact the severity of inflammation, root resorption, and overall treatment duration will enable the development of personalized treatment protocols. Identifying gene polymorphisms that influence OTM outcomes could also predict patients’ responsiveness to specific interventions, such as adjusting orthodontic forces or using genetic-based treatment strategies to mitigate adverse effects.

Biomaterials, Hydrogels, and MMP Inhibitors

In the realm of biomaterials and hydrogels, there is considerable promise in integrating stimuli-responsive materials for the localized and controlled delivery of therapeutic agents during OTM. However, further research is needed to refine methods for ensuring site-specific release, sustained bioactivity, and effective integration of these systems within the periodontal environment. One significant challenge lies in developing hybrid biomaterials that not only promote osteoclastogenesis but also address the need for controlled inflammation and minimal bone resorption. Such biomaterials would hold the potential to enhance treatment outcomes by addressing the dual need for bone remodeling and mitigating unwanted side effects such as root resorption.

MMP inhibitors, such as the FDA-approved Periostat (doxycycline), have shown promise in reducing the proteolytic breakdown of ECM components, preventing excessive destruction during orthodontic treatment. However, the development of more selective MMP inhibitors, such as andecaliximab, which targets MMP-9, could be instrumental in further reducing the adverse effects of OTM, especially in controlling root resorption. While the use of these inhibitors holds potential, additional studies are required to evaluate their efficacy, safety, and long-term impact on orthodontic treatment.

Molecular Therapies and Small Molecules

Molecular and biological therapies represent a growing field for the modulation of inflammation and osteoclastogenesis during OTM. Identifying molecular targets that can selectively modulate these processes could reduce unwanted side effects such as external root resorption while promoting efficient tooth movement. For example, small molecules, biologics, or gene-editing technologies that fine-tune the balance between osteoclast and osteoblast activity could optimize bone remodeling during treatment. Research must aim to identify safe, effective molecular therapies that can be incorporated into orthodontic care to improve treatment predictability, reduce side effects, and enhance overall treatment outcomes.

One promising molecular approach involves the use of plant-based rhCol1, which has been shown to support PDL regeneration. Studies have demonstrated the potential of rhCol1 to facilitate the reconstruction of the PDL matrix following force-induced destruction. Further research is needed to optimize the use of rhCol1 and other collagen-based materials in enhancing PDL regeneration, and to explore their synergy with other treatments such as growth factors and cytokines, which could accelerate healing and enhance orthodontic outcomes.

Additionally, the use of Cilengitide, an RGD sequence mimetics, offers a novel strategy to prevent the adhesion of osteoclasts and odontoclasts to the ECM of alveolar bone and root cementum, potentially reducing unwanted bone resorption during orthodontic treatments. By inhibiting integrin binding, Cilengitide has shown promise in blocking osteoclastogenesis and odontoclastogenesis, thus preserving bone integrity. Incorporating such targeted molecular interventions could further improve treatment outcomes by controlling bone resorption and enhancing tissue preservation.

Artificial Intelligence and Personalized Treatment

AI presents a tremendous opportunity to revolutionize orthodontic treatment by enabling more personalized and data-driven care. However, current AI models require refinement to fully integrate patient-specific variables, such as genetic factors, bone density, and individual inflammatory responses. Future research should focus on advancing AI algorithms to predict patient-specific orthodontic responses with greater accuracy, allowing for tailored treatment plans that adapt to a patient’s unique biological conditions.

AI could also play a significant role in real-time monitoring and adjustment of orthodontic treatments based on physiological feedback, such as biomarkers for bone remodeling or cellular responses. By incorporating dynamic, real-time data into treatment planning, AI could allow for continuous optimization of orthodontic care, improving the speed and efficiency of tooth movement while minimizing side effects such as root resorption.

Interdisciplinary Collaboration and Integration

The integration of cellular therapies, biomaterials, molecular interventions, and AI into clinical practice will require interdisciplinary collaboration and continued innovation. Researchers from diverse fields such as cell biology, biomaterials engineering, molecular genetics, and AI will need to work together to address these open research questions. As a result, they can develop safe, effective, and personalized treatment approaches that will shape the future of orthodontic care.

Addressing these open research questions will be essential not only for improving the predictability and safety of orthodontic procedures but also for enabling a more tailored, efficient, and scientifically grounded approach to orthodontic treatment. As our understanding of gene polymorphisms, molecular pathways, and advanced technologies deepens, we can expect to see significant improvements in both the outcomes and the patient experience in orthodontics.

## 4. Integrating Cellular, Molecular, and Digital Innovation to Shape the Future of Orthodontic Treatment

Orthodontics is experiencing a transformative shift driven by innovations in cellular biology, biomaterials, molecular therapies, and artificial intelligence (AI). This review has highlighted how these technologies can influence and reshape orthodontic treatment, providing new avenues for personalized and more efficient care. However, as evidenced in recent research, the integration of these cutting-edge technologies into clinical orthodontic practice faces both exciting opportunities and significant challenges.

Cellular and regenerative approaches, especially those involving stem cells, hold great promise for enhancing orthodontic treatments. iPSCs have shown potential for accelerating bone remodeling during OTM by differentiating into osteoclasts and osteoblasts [102]. However, challenges related to the safety, scalability, and potential tumorigenesis of these cells must be addressed before they can be safely applied in clinical orthodontics [154]. Additionally, the ability to control stem cell differentiation and delivery systems is crucial for optimizing their use in orthodontic settings.

Gene polymorphisms also play a pivotal role in orthodontic outcomes, particularly in relation to inflammatory responses and bone remodeling during OTM. Polymorphisms in genes like IL-1β (and IL-1β in conjunction with IL1RN) have been linked to increased susceptibility to root resorption [49], offering a potential avenue for personalized orthodontic care. The identification of genetic markers for complications such as external apical root resorption (OEARR) can help predict a patient’s response to orthodontic treatment and guide individualized treatment plans. Similarly, genetic markers associated with the duration of treatment, such as those identified in recent studies [79], could enable clinicians to better estimate treatment time and optimize therapeutic strategies based on the patient’s genetic predisposition.

In parallel, biomaterials such as hydrogels and collagen-based carriers have shown promise in facilitating localized drug delivery and tissue regeneration during OTM. Hydrogels responsive to mechanical stimuli and capable of releasing bioactive agents can promote osteoclastogenesis and accelerate tooth movement [155,156]. For example, plant-based rhCol1 and collagen-based hydrogels have demonstrated success in regenerating the PDL matrix and promoting faster recovery from hyalinization [94,95]. However, optimizing the degradation rates and bioactivity of these materials is essential to ensure that they deliver therapeutic agents at the right time and location, without triggering excessive inflammation. Hybrid materials that support osteoclast and osteoblast activity while integrating seamlessly with surrounding tissues offer another promising direction [157,158,159].

The use of Cilengitide, an RGD sequence mimetic, represents a promising molecular approach to mitigate osteoclast and odontoclast activity during orthodontic treatments. By mimicking the Arg-Gly-Asp (RGD) sequence, Cilengitide competitively inhibits integrin binding to the extracellular matrix (ECM) of alveolar bone and root cementum, preventing the adhesion of osteoclasts and odontoclasts. Recent studies have demonstrated the potential of Cilengitide in reducing osteoclast differentiation and activity in both in vitro and in vivo models [106,160]. Specifically, it has been shown to inhibit osteoclastogenesis and protect against root resorption during orthodontic tooth movement, making it a promising therapeutic tool to reduce bone resorption and preserve the integrity of the alveolar bone and root cementum. Incorporating such targeted molecular therapies could enhance the efficacy of orthodontic treatment by controlling unwanted side effects related to excessive bone resorption.

MMP inhibitors, particularly doxycycline (Periostat), have been shown to reduce the destructive effects of proteolytic activity during OTM, mitigating excessive bone resorption and thus minimizing the risk of OEARR [85].

More selective inhibitors targeting MMP-9, such as andecaliximab, offer targeted therapies to reduce unwanted side effects, since their safety, pharmacokinetics and disease-related outcome was evaluated in clinical studies [86].

These strategies could be particularly useful in managing the complications associated with excessive bone resorption and inflammatory responses during orthodontic treatments.

Small molecules, such as PROTACs, represent an exciting frontier in molecular therapies. PROTACs are designed to induce the degradation of specific proteins, offering a highly targeted approach to modulating the signaling pathways involved in osteoclastogenesis and osteogenesis. By selectively targeting and degrading proteins involved in excessive bone resorption, PROTACs, e.g., directed against osteoclastogenesis-triggering RANKL, could potentially minimize the negative effects of OTM while enhancing the desired outcomes [161]. However, the development and optimization of PROTACs for use in orthodontics require further research to assess their specificity, safety, and long-term effects.

The molecular therapies aimed at modulating cytokine and growth factor signaling pathways, such as RANKL and OPG, are also promising. These pathways regulate osteoclastogenesis and osteogenesis, playing a crucial role in the balance between bone resorption and formation during OTM [162]. Fine-tuning these molecular interventions offers the potential to improve treatment outcomes and reduce complications. However, the complexity of these signaling pathways and the need for precise modulation remain significant challenges. More research is needed to understand how to balance the activation of these pathways without triggering unwanted side effects, such as excessive bone loss or root resorption.

AI is rapidly becoming an integral part of orthodontics, particularly in treatment planning, personalized care, and predictive modeling. AI-driven systems, which are based on deep learning algorithms, can analyze large datasets [163] such as radiographs and 3D imaging to identify patient-specific factors that could influence treatment outcomes [152]. Recent studies have also shown that AI can predict tooth movement patterns based on genetic and phenotypic data, further enhancing treatment personalization [151]. In the context of personalization, it should be noted that a deep learning framework has for instance been used for individualizing radiotherapy dose [153].

However, to fully realize the potential of AI in orthodontics, it is essential to develop real-time AI systems capable of adjusting treatment plans dynamically as the patient progresses [164]. By integrating AI with molecular therapies and biomaterials, orthodontic treatment could become more personalized and efficient.

Despite the promise of these technologies, several barriers must be overcome to ensure their clinical adoption. Regulatory approval of biologics and stem cell-based therapies remains a significant hurdle, particularly given the challenges of demonstrating their long-term safety and efficacy [165]. Ethical considerations surrounding the use of gene-editing technologies and stem cell therapies must also be addressed to ensure their safe and responsible application in orthodontic care. Moreover, concerns regarding patient privacy, especially with AI systems handling sensitive data, must be resolved to foster trust in these technologies.

In conclusion, the future of orthodontics lies in the integration of advanced cellular, molecular, and AI-driven technologies. These innovations offer the potential to significantly enhance the personalization, efficiency, and safety of orthodontic treatments. Continued research and development in gene polymorphisms, stem cell therapies, biomaterials, small molecules like PROTACs, and AI-driven systems will be crucial for shaping the next generation of orthodontic care. However, their successful clinical implementation will require overcoming significant regulatory, ethical, and technical challenges to ensure that these promising technologies can be safely and effectively incorporated into routine orthodontic practice.

### Clinical Translation: Opportunities, Limitations, and Challenges

While the conceptual advances discussed in this review—ranging from iPC-derived osteoclast delivery to monoclonal antibodies and miRNA (miR)-based diagnostics—hold transformative potential, several critical barriers must be overcome before these strategies can be routinely implemented in clinical orthodontics.

First, regulatory complexity poses a substantial translational hurdle. Therapeutics involving gene editing (e.g., miR modulation), targeted protein degradation (e.g., PROTACs), or patient-specific stem cell products (e.g., iPC-derived osteoclasts) must undergo rigorous approval processes governed by regulatory agencies such as the FDA or EMA. These challenges are compounded by safety concerns surrounding stem cell differentiation efficiency, tumorigenesis risk, and off-target effects [154,165].

Second, economic feasibility must be considered. The generation and validation of iPCs for individual patients or the performance of predictive miR screenings remain cost-intensive and technologically demanding. Similarly, the clinical use of biologics such as denosumab or infliximab, while supported by existing safety data in systemic diseases, introduces substantial financial burden and would likely be limited to high-risk cases [115,127].

Third, ethical and privacy-related concerns are emerging, especially when considering pre-therapeutic genotyping or AI-assisted treatment planning based on omics data. The integration of genetic markers—such as IL-1 or P2RX7 polymorphisms—into treatment decisions must adhere to strict guidelines on informed consent and data governance, particularly under legislation such as the European General Data Protection Regulation (GDPR) [152,164].

Fourth, clinical integration and feasibility must be pragmatically evaluated. Tools such as AI-assisted aligner planning or injectable RANKL hydrogels require real-world validation in terms of cost-effectiveness, user-friendliness, and compliance. For example, topical MMP inhibitors or cytokine-loaded scaffolds may require repeat applications or customization per patient-specific inflammatory status, which may complicate standardized workflows [85,96].

In summary, while the interdisciplinary innovations described in this review hold promise for the future of precision orthodontics, their clinical implementation will depend on comprehensive assessment of safety, regulatory approval, cost-effectiveness, ethical acceptability, and integration into everyday clinical practice.

## 5. Conclusions

The future of orthodontics lies in a multidisciplinary approach that combines cellular therapies, biomaterials, molecular biology, and artificial intelligence. These advancements, while promising, necessitate significant further research to overcome current limitations, including the safety and regulatory hurdles surrounding stem cell use, the optimization of biomaterials for localized delivery, and the fine-tuning of molecular therapies for precise modulation of bone remodeling.

As the field moves forward, collaboration between orthodontists, bioengineers, molecular biologists, and AI experts will be crucial to drive innovation and address open research questions to shape prospective orthodontics. Clinical trials that investigate the long-term outcomes of combining these technologies, as well as the development of new AI-driven treatment protocols, will be critical in establishing these innovations as reliable tools for personalized orthodontic care. Ultimately, the integration of these cutting-edge technologies could lead to more efficient, less invasive, and more predictable orthodontic treatments, significantly improving patient outcomes in the coming decades.

## Figures and Tables

**Table 1:** Summary of stress and inflammation in early OTM.

Pathway/ Factor	Role in OTM	Mechanism	Implication	References
Hypoxia/HIF-1α	Triggers cell death	Mitochondrial apoptosis via Bax, caspase cascade	Zone clearance delay (lag phase)	[9,10,11]
MMPs (MMP-8, -9, -13)	ECM degradation	Collagen proteolysis	Hyalinization, loss of PDL structure	[13,14,38]
IL-1β, TNF-α, IL-6	Drive sterile inflammation	TLR4 → MyD88/IRAK/TRAF6 → NF-κB	Stimulate osteoclastogenesis, pain response	[28,29,30,32]
TIMPs (TIMP-1/2/4)	Inhibit MMPs	Protease inhibition	Potential target to balance matrix turnover	[39,41,42,43]

**Table 3:** RANK/RANKL/OPG Axis in OTM.

Component	Function	Regulators	Impact	References
RANKL	Induces osteoclast fusion/differentiation	TNF-α, IL-1β, miR-125a	Bone resorption, OTM progression	[48,56,57]
RANK	Osteoclast precursor receptor	RANKL, miR-125a	Activates NF-κB, c-Fos, NFATc1	[52,56]
OPG	Decoy receptor, inhibits RANKL-RANK	miR-3198 (represses OPG)	Protective against bone loss/OEARR	[62,63,64]

**Table 4:** Polymorphisms linked to inflammation and OEARR risk. Arrow means increased inflammatory risk.

Gene	Polymorphism	Effect	Clinical Relevance	References
IL-1β	+3954 C/T	Elevated IL-1β in GCF, ↑ inflammatory risk	Higher OEARR susceptibility	[76,78]
P2RX7	rs208294	Impaired ATP receptor function	Strongly linked to root resorption	[49,50]
IL-1RN	VNTR alleles	Alters IL-1 antagonist levels	May exacerbate inflammatory cascades	[78]

**Table 5:** Approaches to PDL regeneration after hyalinization. Arrow means increased NO level, leading to vasodilation and increased perfusion.

Intervention	Mechanism	Clinical Benefit	References
rhCol1 + BMP hydrogels	Matrix replacement + growth factor delivery	PDL matrix regeneration	[94,95]
MMP inhibitors (Periostat)	Blocks ECM degradation	Shortens lag phase, preserves collagen	[85,86]
Arginine/Citrulline	↑ NO → vasodilation → ↑ perfusion	Enhanced macrophage activity and matrix repair	[89]
Periostin + CCN2	Stimulates fibroblast migration and adhesion	Speeds wound healing	[90,91,92]

**Table 6:** RANKL and iPC-based hydrogel strategies.

Component	Function	Advantage	Limitation	References
RANKL Hydrogels	Stimulate osteoclast activity	Spatio-temporal control of bone resorption	Requires optimal dosing and biocompatibility	[96,97,98,101]
iPC-derived Osteoclasts	Personalized resorption units	Non-invasive, patient-specific therapies	Safety, regulatory barriers	[99,100,102]

**Table 7:** Strategies to prevent or reverse root resorption.

Strategy	Mechanism	Target	References
Cilengitide	Blocks integrin-mediated adhesion	Osteoclasts/Odontoclasts	[51,105,106,107]
Biomimetic Cementum	Artificial cementum regeneration	Cementoblasts	[104]
Cytokine Inhibition	IL-1R/IL-6R blockade reduces osteoclastogenesis	Inflammatory response	[108,109]

**Table 8:** PROTAC applications in inflammatory modulation during OTM.

Target	Degraded Protein	Mechanism	Potential Use in OTM	References
NF-κB	p65/p50 subunits	Ubiquitin–proteasome degradation	Reduces RANKL induction	[110,111,112]
PROTAC uptake	Folate/HER2-coupled	Targeted intracellular delivery	Improves selectivity	[111,114]

**Table 10:** AI-based tools in orthodontics: validated vs. experimental strategies.

Application Area	Validated AI Tools	Experimental/Research-Phase AI Models
Cephalometric analysis	CephX^®^ (Orca Dental AI Ltd., Tel-Aviv, Israel), Vatech EzOrtho AI (Vatech Co., Ltd. Hwaseong-si, Republic of Korea, WebCeph™ (AssembleCircle Corp., Seongnam-si, Republic of Korea [137,138]	Generative adversarial networks (GANs) for automated landmark detection [139]
Treatment planning	3Shape OrthoAnalyzer™ AI (3Shape A/S, Copenhagen, Denmark),(aligner sequencing), Dolphin Imaging AI (Patterson Dental (Dolphin Imaging & Management Solutions) Chatsworth, CA, USA [140]	Reinforcement learning for force system optimization [141]
Tooth segmentation	Deep learning CNNs for automated dental arch segmentation (FDA-cleared) [142]	Multimodal segmentation using fused intraoral scans and CBCT [143]
Outcome prediction	Aligner tracking algorithms based on validated movement thresholds [144]	Predictive AI models for treatment duration and risk of OEARR using EMR + radiomics [145]

**Table 11:** Applications of AI in OTM and orthodontic care.

Application	Function	Clinical Benefit	References
Virtual Twin Modeling	Simulates jaw/teeth movement	Precise appliance design	[148,150]
Predictive Treatment AI	Estimates treatment response based on data	Shorter duration, fewer side effects	[151,152]
Diagnostic AI Imaging	Detects malocclusion/pathology in radiographs	Enhanced diagnostic sensitivity	[152,153]

## Data Availability

Not applicable.

## Abbreviations

ACS	Acute Coronary Syndrome
AKT	Protein Kinase B
ALP	Alkaline Phosphatase
AP-1	Activator Protein 1
ATP	Adenosine Triphosphate
Bcl6	B-cell Lymphoma 6
BMP2	Bone Morphogenetic Protein 2
CCL17	Chemokine (C-C Motif) Ligand 17
CCL22	Chemokine (C-C Motif) Ligand 22
CD36	Cluster of Differentiation 36
CD80	Cluster of Differentiation 80
CD86	Cluster of Differentiation 86
CD200R	Cluster of Differentiation 200 Receptor
CD206	Cluster of Differentiation 206
COL1	Collagen Type I
COL2	Collagen Type II
COL3	Collagen Type III
COL4	Collagen Type IV
COL5	Collagen Type V
CXCL10	C-X-C Motif Chemokine Ligand 10
CXCL11	C-X-C Motif Chemokine Ligand 11
CYLD	Cylindromatosis
ECM	Extracellular Matrix
ERK	Extracellular Signal-Regulated Kinase
FAK	Focal Adhesion Kinase
GSK3β	Glycogen Synthase Kinase 3 Beta
HSP	Heat Shock Protein
IKK	IκB Kinase
IL-1β	Interleukin-1 Beta
IL-6	Interleukin-6
IL-8	Interleukin-8
IL-10	Interleukin-10
IL-12	Interleukin-12
IL-23	Interleukin-23
IL-34	Interleukin-34
JAK	Janus Kinase
LPS	Lipopolysaccharide
MHCII	Major Histocompatibility Complex II
miR-125a	MicroRNA-125a
MMP-1	Matrix Metalloproteinase 1
MMP-8	Matrix Metalloproteinase 8
MMP-9	Matrix Metalloproteinase 9
MMP-13	Matrix Metalloproteinase 13
mTOR	Mechanistic Target of Rapamycin
MyD88	Myeloid Differentiation Primary Response 88
NF-κB	Nuclear Factor Kappa B
NFATc1	Nuclear Factor of Activated T-Cells 1
NEMO	NF-κB Essential Modulator
OSX	Osterix
p38	p38 Mitogen-Activated Protein Kinase
PDL	Periodontal Ligament
PROTAC	Proteolysis-Targeting Chimera
Rac1	Ras-Related C3 Botulinum Toxin Substrate 1
RANK	Receptor Activator of NF-κB
RANKL	Receptor Activator of NF-κB Ligand
RNA	Ribonucleic Acid
ROS	Reactive Oxygen Species
RUNX2	Runt-Related Transcription Factor 2
SEMA3A	Semaphorin 3A
SOST	Sclerostin
TGF-β	Transforming Growth Factor Beta
TIMPs	Tissue Inhibitors of Metalloproteinases
TLR4	Toll-Like Receptor 4
TNF-α	Tumor Necrosis Factor Alpha
TRAF6	TNF Receptor-Associated Factor 6
TRAP	Tartrate-Resistant Acid Phosphatase
VEGF	Vascular Endothelial Growth Factor
